# Interactions of Trivalent Lanthanide Cations with a New Hexadentate Di-Schiff Base: New Lanthanide(III) Complexes from (NE,N′E)-2,2′-(ethane-1,2-diylbis(oxy))bis(N-(pyridin-2-ylmethylene)ethanamine)

**DOI:** 10.1155/2010/613140

**Published:** 2010-06-22

**Authors:** Mantha Tsiouri, Konstantina Skorda, Christos Papadimitriou, Yang Li, J. Derek Woollins, John C. Plakatouras

**Affiliations:** ^1^Department of Chemistry, University of Ioannina, 451 10 Ioannina, Greece; ^2^Faculty of Physics and Chemistry, Hellenic Army Academy, 166 73 Vari, Greece; ^3^School of Chemistry, University of St. Andrews, North Haugh, St. Andrews, Fife, Scotland, KY16 9ST, UK

## Abstract

The novel lanthanide(III) complexes [Ln(NO_3_)_2_L](NO_3_)·3MeOH (Ln = La **1**, Pr **2**) and [Ln(NO_3_)_3_L](NO_3_)·2MeOH (Ln = Gd **3**, Yb **4**), where L = (NE,N′E)-2,2′-(ethane-1,2-diylbis(oxy))bis(N-(pyridin-2-ylmethylene)ethanamine), have been obtained by direct reaction of the Schiff base ligand and the corresponding hydrated lanthanide(III) nitrates in methanol. All complexes were characterized spectroscopically and thermogravimetrically. Complex **4** was also characterized with crystallographic studies: orthorhombic P2_1_2_1_2_1_, *a* = 10.6683(14), *b* = 13.4752(15), *c* = 19.3320(26) Å. In the molecular structure of **4**, Yb(III) is surrounded by all donor atoms of the Schiff base (four nitrogen and two oxygen atoms) and four oxygen atoms belonging to two bidentate chelating nitrato ligands.

## 1. Introduction

Schiff base metal complexes have a key role in the development of coordination chemistry,resulting in an enormous number of publications, ranging from pure synthetic work to modern physicochemical and biochemically relevant studies of metal complexes [1].

The lanthanide cations can promote Schiff base condensation and can give access to complexes of otherwise inaccessible ligands. This fact, in combination with the applications of lanthanide macrocyclic complexes emerging from biology and medicine, has boosted research on these areas [2]. 

One of the major applications of lanthanide complexes in medicine is their use as water proton relaxation agents for NMR imaging [3, 4]. The research in this field is directed towards the synthesis of stable, nontoxic, highly paramagnetic molecules with the ability to improve efficiently the contrast of the magnetic resonance image. The number of coordinated water molecules on the paramagnetic center (usually gadolinium(III)) greatly contributes to the relaxivity (the efficiency with which the complex enhances the proton relaxation rates of water) of the contrast agent. Initially polyaminocarboxylates were utilized as ligands for the preparation of such complexes, with [Gd(DTPA)(H_2_O)]^2-^ (DTPAH_5_ = diethylenetriaminepentaacetic acid) being the most commonly used contrast agent. Though the aforementioned agent has a high stability constant, reducing the toxic effect of the free metal ion, its disadvantage is the availability of only one water coordination site.

A large number of articles have been published on lanthanide complexes with the hexadentate Schiff base derived by the condensation of 2,6-diacetylpyridine and ethylenediamine [5–16]. These complexes are stable enough under physiological conditions. However, only recently, research work dealing with the various aspects involving different physicochemical properties and complexation behaviour of tetradentate Schiff bases has appeared in the literature, primarily focusing on the separation of actinides from lanthanides in nuclear reprocessing [17] and catalytic properties [18]. 

Previously, we have reported the synthesis and the structural and spectroscopic characterization of lanthanide complexes with N,N′-bis[1-(pyridin-2-yl)ethylidene]ethane-1,2-diamine [19] and N,N′-bis(pyridin-2-yl-methylene)benzene-1,2-diamine [20]. It was found that the ethylenediamine “hinge” of the di- Schiff base ligand eliminates the possibility of coplanar coordination of the four nitrogen donors [19] while when we changed the ethytlene diamine moiety with 1,2-phenylenediamine to force planar coordination of the tetradentate di-Schiff base, we ended up with the lanthanide cations outside of the four nitrogens plane [20]. In a different work, [21], we found that N,N′-bis(pyridin-2-ylmethylene)cyclohexane-1,2-diamine has an intermediate coordinating behaviour to the lanthanides. 

In a previous work, the utilization of 8-hydroxyquinoline-2-carboxaldehyde for the preparation of Schiff bases leads to products with poor solubility [22]. The next step in our research is to elongate the diamine part of the above-mentioned ligands, incorporating donor atoms, and study the structure and the stability of the prepared complexes. Since the denticity of the ligands is increased, we expected more stable complexes. Herein we report the synthesis and characterization of four new lanthanide complexes with (NE,N′E)-2,2′-(ethane-1,2-diylbis(oxy))bis(N-(pyridin-2-yl-methylene)ethanamine), a ligand derived by the condensation of 2,2′-(ethane-1,2-diylbis(oxy))diethanamine and pyridine-2-carboxaldehyde.

## 2. Experimental

### 2.1. Materials and Instrumentation

All manipulations were carried out under aerobic conditions. Metal salts and organic molecules were purchased from Aldrich and used as received. Solvents were of analytical grade (Lab-Scan Chemical Co) and used without further purification. C, H, and N analyses, IR (4000–370 cm^−1^) and far-IR (600–30 cm^−1^) [20], and UV/Vis spectra in the solid state [23] and in solution, [24] thermal studies, and room temperature magnetochemical measurements [20] were carried out as previously described.

### 2.2. Preparation of the Compounds

The ligand (NE,N'E)-2,  2′-(ethane-1,2-diylbis(oxy))bis(N-(pyridin-2-ylmethylene)-ethanamine) was synthesized from the condensation of 2,2′-(ethane-1,2-diylbis(oxy))diethanamine and pyridine-2-carboxaldehyde *in situ*. 

To a stirred solution of pyridine-2-carboxaldehyde (0.40 g, 3.73 mmol) in methanol (15 mL), a solution of 2,  2′-(ethane-1,2-diylbis(oxy))diethanamine (0.27 g, 1.86 mmol) in methanol (5 mL) was added. The resulting yellowish solution was refluxed for 1 hr, and to this the corresponding hydrated lanthanide nitrate (1.86 mmol) in 10 ml of methanol was added. The resulting solutions were heated for a further 30 min and then was left undisturbed to evaporate at room temperature, and yielded microcrystalline solids after three days. The solids were isolated by filtration, washed with a small amount of cold methanol (*ca*. 2 mL) and diethyl ether (2 × 10 mL), and dried under vacuum, over silica gel. The yields were within the range of 55%–65%. A small portion of the mother liquid of complex **4** was layered with diethyl ether to yield a few small colourless blocks, suitable for X-ray structural studies. 

[La(NO_3_)_2_L](NO_3_)·3MeOH (**1**) Anal. Calc. for C_23_H_34_N_7_O_14_La: C, 35.80; H, 4.45; N, 12.71. Found: C, 35.96; H, 4.09; N, 12.57%. Selected IR data (cm^−1^): 3377 mw [m(O–H)], 3070 w [m(C–H)_ar_], 2980 w [m(C–H)_al_], 1759 m, 1733 m [*ν*_1_ + *ν*_4_ of the nitrate], 1641 s [m(C=N)], 1590 ms, 1572 m [ring stretching vibrations], 1492 versus [*ν*_1_(A_1_) of the nitrate], 1309 versus [*ν*_5_(B_2_) of the nitrate], 1018 m [*ν*_2_(A_1_) of the nitrate], 636 m [*δ*(py)], 410 w [*γ*(py)]. TGA/DTA (N_2_, 1 atm): 50°C–91°C (–MeOH, found: 12.81, cald.: 12.46%, endotherm), 245°C–380°C (decomposition, sharp exotherm), 591°C (final plateau, La_2_O_3_, found: 20.91, cald.: 21.11%). 

[Pr(NO_3_)_2_L](NO_3_)·3MeOH (**2**) Anal. Calc. for C_23_H_34_N_7_O_14_Pr: C, 35.71; H, 4.44; N, 12.68. Found: C, 35.63%; H, 4.00%; N, 12.39%. Selected IR data (cm^−1^): 3389 mw [m(O–H)], 3069 w [m(C–H)_ar_], 2981 w [m(C–H)_al_], 1762 m, 1731 m [**ν**_1_ + **ν**_4_ of the nitrate], 1645 s [m(C=N)], 1592 ms, 1571 m [ring stretching vibrations], 1490 versus [*ν*_1_(A_1_) of the nitrate], 1311 versus [*ν*_5_(B_2_) of the nitrate], 1020 m [*ν*_2_(A_1_) of the nitrate], 637 m [**δ**(py)], 406 w [**γ**(py)]. *μ*_eff_ = 3.47 BM at 21°C. TGA/DTA (N_2_, 1 atm): 61°C–92°C (–MeOH, found: 12.01, cald.: 12.43%, endotherm), 257°C–337°C (decomposition, sharp exotherm), 547°C (final plateau, Pr_6_O_11_, found: 21.99, cald.: 22.01%).

[Gd(NO_3_)_2_L](NO_3_)·2MeOH (**3**) Anal. Calc. for C_22_H_30_N_7_O_13_Gd: C, 34.86; H, 4.00; N, 12.94. Found: C, 34.87; H, 3.72; N, 13.00%. Selected IR data (cm^−1^): 3386 mw [m(O–H)], 3070 w [m(C–H)_ar_], 2982 w [m(C–H)_al_], 1767 m, 1734 m [*ν*_1_  +  *ν*_4_ of the nitrate], 1642 s [m(C=N)], 1590 ms, 1573 m [ring stretching vibrations], 1488 versus [*ν*_1_(A_1_) of the nitrate], 1310 versus [*ν*_5_(B_2_) of the nitrate], 1030 m [*ν*_2_(A_1_) of the nitrate], 630 m [**δ**(py)], 417 w [**γ**(py)]. *μ*_eff_ = 7.96 BM at 21°C. TGA/DTA (N_2_, 1 atm): 51°C–70°C (–MeOH, found: 8.98, cald.: 9.12%, endotherm), 283°C–345°C (decomposition, sharp exotherm), 508°C (final plateau, Gd_2_O_3_, found: 23.57, cald.: 23.92%).

[Yb(NO_3_)_2_L](NO_3_)·2MeOH (**4**) Anal. Calc. for C_22_H_30_N_7_O_13_Yb: C, 34.15; H, 3.92; N, 12.68. Found: C, 34.17; H, 3.77; N, 13.59%. Selected IR data (cm^−1^): 3401 mw [m(O–H)], 3078 w [m(C–H)_ar_], 2967 w [m(C–H)_al_], 1762 m, 1738 m [*ν*_1_  +  *ν*_4_ of the nitrate], 1633 s [m(C=N)], 1581 ms, 1562 m [ring stretching vibrations], 1497 versus [*ν*_1_(A_1_) of the nitrate], 1308 versus [*ν*_5_(B_2_) of the nitrate], 1039 m [*ν*_2_(A_1_) of the nitrate], 618 m [**δ**(py)], 422 w [**γ**(py)]. *μ*_eff_ = 7.96 BM at 21°C. TGA/DTA (N_2_, 1 atm): 48°C–71°C (–MeOH, found: 8.45, cald.: 8.80%, endotherm), 288°C–361°C (decomposition, sharp exotherm), 545°C (final plateau, Yb_2_O_3_, found: 25.01, cald.: 25.47%).

### 2.3. X-Ray Crystallography

X-ray crystal data (Table 1) were collected at 93 K by using a Rigaku MM007 High brilliance RA generator/confocal optics and Mercury CCD system. Intensities were corrected for Lorentz polarization and for absorption. The structure was solved by direct methods [SIR-97]. [25] Hydrogen atoms bound to carbon were idealised. Structural refinement was obtained with full-matrix least-squares based on *F^2^*  by using the program SHELXL. [26] The crystal was a racemic twin and refined smoothly using TWIN and BASF commands incorporated in SHELX. CCDC 776362 contains the supplementary crystallographic data for this paper. These data can be obtained free of charge via http://www.ccdc.cam.ac.uk/conts/retrieving.html or from the Cambridge Crystallographic Data centre, 12 Union Road, Cambridge CB2 1EZ, UK; fax (+44) 1223-336-033; E-mail: deposit@ccdc.cam.ac.uk.

## 3. Results and Discussion

**Scheme 1:**
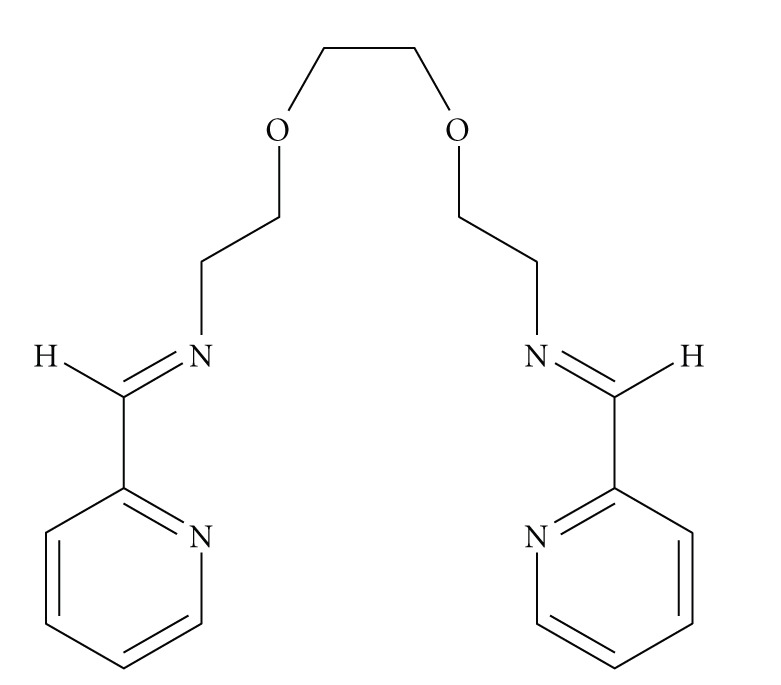
The ligand (NE,N'E)-2,2′-(ethane-1,2-diylbis(oxy))bis(N-(pyridin-2-yl-methylene)ethanamine) (L).

Complexes **1**–**4** were prepared by direct reaction of the hydrated lanthanide nitrate salts and the Schiff base ligand starting materials in methanol in 1 : 1: 2 metal to diamine to aldehyde molar ratio. Though it seems that the ligand (Scheme 1) can be prepared by the direct reaction of its constituents (2,  2′-(ethane-1,2-diylbis(oxy))diethanamine and pyridine-2-carboxaldehyde in 1 : 2 molar ratio), it was impossible to isolate it in a solid form. This is probably due to the relatively long dietheric chain between the Schiff base moieties. Attempts to prepare the 1 : 2 complexes using larger excess of the ligand parts and different solvents lead to impure products with unidentified formulae.

**Figure 1:**
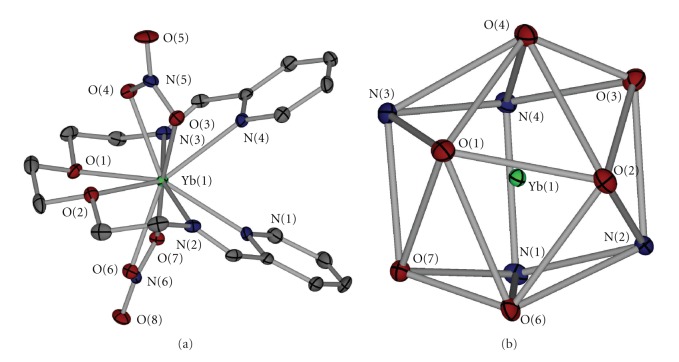
(a) A thermal ellipsoid plot of the cation in **4**, including a partial labelling scheme. The hydrogen atoms have been omitted for clarity. (b) The coordination polyhedron about Yb(1) in **4**.

The crystal structure of complex **4** (Figure 1(a)) consists of cationic complexes [Yb(NO_3_)_2_L]^+^, nitrate anions, and two methanol molecules per metal ion held together with hydrogen bonds and C–H⋯*π* interactions. The Yb^III^ atom, being ten coordinated, is surrounded by six oxygen atoms belonging to two bidentate chelating nitrato ligands and to the etherate part of the ligand and four (two imino and two pyridine) nitrogen atoms belonging to the Schiff base ligand. The coordination polyhedron is much distorted and can be better described, according to Robertson [27], being between a Hoard dodecahedron and a decatetrahedron. (Figure 1(b)).

The Yb–O bond distances span the range 2.424–2.543 Å and they are in good agreement with previously reported values [7, 8, 17–21, 28], taking into account the lanthanide contraction. Though neutral, the etheric oxygen atoms appear to be coordinated to Yb^III^ stronger than the nitrates (mean Yb–O_nitrato_ = 2.47, Yb–O_ether_ = 2.43 Å). There are two probable reasons for this behavior: (a) the macrochelate effect, since the ligand contains six sequential donor atoms and (b) the coordination behavior of the nitrato ligands which are both assymetrically chelated. There are no important differences in the coordination characteristics of the nitrato ligands. Additionally, both of the coordinated nitrates are coplanar to Yb(1), as indicated by the Yb(1)–O_coordinated_–N–O_free_ torsion angles which are all larger than 175°C. The mean Yb–N distance is 2.478 Å, with the Yb–N_imino_ being shorter than the corresponding Yb– N_pyridine_ and is in agreement with our previous data [19–21]. 

**Scheme 2:**
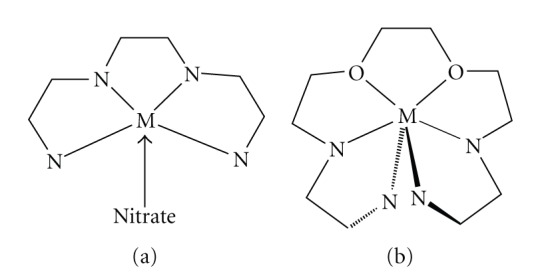
A schematic representation of the coordination of the Schiff base ligands used, to lanthanides. (a) References 18–21, (b) This work.

The major differences from our previous work on lanthanoid coordination chemistry with Schiff bases rise from the size and the denticity of the ligand. When tetradentate N_4_ ligands were used, (Scheme 2(a)) the four nitrogen donor atoms are coplanar and the metal ion lies approximately on the plane formed. There is also space for a nitrato ligand to approach and bind the lanthanide cation. In the present case, the two chelating pyridine Schiff base coordination sites exist, but their distances have been significantly increased. In addition, between the nitrogen chelating sites are present two more oxygen donor atoms which eventually bind the lanthanide. (Scheme 2(b)) Furthermore, there is no space to accommodate six donor atoms on the same plane about Yb^III^ and this results in the exclusion of the pyridine nitrogen away of the plane formed (max deviation from the mean plane: 0.051 Å) by the two oxygen and the two imino nitrogen atoms. This way the two pyridine rings are pointing up and down of the plane with a dihedral angle of 53.846(5)°. With the tetradentate Schiff bases being coordinated to lanthanides, there is enough room for three nitrato ligands to coordinate to the metal. Increasing the denticity of the ligand to six, only two nitrato ligands appear in the coordination sphere of the metal. 

The ionic nitrate interacts with H-bonds with the solvated methanol molecules. Surprisingly, the coordinated nitrates are not involved in hydrogen bonding interactions. This is probably due to their location in cavities with organic surfaces in the crystal.

Comparison of the infrared spectra of the prepared complexes can lead us rather safely to the conclusion that the four complexes are isostructural or at least that both the nitrato and the Schiff base ligands are coordinated in the same manner. Assignment of the nitrate bands has been made as reported previously [29].

The room temperature effective magnetic moments of complexes **2**, **3**, and **4** show little deviation from the Van Vleck theoretical values.

The values of the bonding parameters *β* (nephelauxetic ratio) and *δ* (Sinha's parameter) of the Pr^III^ complex 2 are calculated from the solid state *f–f* spectra by standard equations. Those are 1.003 and −0.30, respectively, and they suggest that the interaction between the trivalent lanthanide and the ligands is essentially electrostatic and that there is very small participation of the 4*f* orbitals in bonding [30].

All complexes behave similarly when heated under nitrogen. Complexes **1** and **2** lose their solvated methanol molecules above its boiling point, while **3** and **4** lose the solvated molecules about methanol's boiling point. This is an indication of stronger H–bonding in their structures. The intermediates are reasonably stable (up to ca. 280°C) and decompose violently due to the nitrates. The final residues, which are obtained above 490°C, correspond to the sesquioxides except for **2**, which corresponds to Pr_6_O_11_.

Preliminary relaxometric data have shown that **3** is pretty stable in aqueous medium in addition to its increased solubility due to the ionic character. Tailoring, with major goal to increase the stability of lanthanide complexes, of new ligands based on the Schiff base described here and previously [18–22] is in progress.

## Figures and Tables

**Table 1:** Crystal data and structure refinement for 4.

	[Yb(NO_3_)_2_L](NO_3_)·2MeOH
Empirical formula	C_20_H_30_N_7_O_13_Yb
Formula weight	749.55
Temperature	93(2) K
Wavelength	0.71069 Å
Crystal system, Space group	Orthorhombic, P2_1_2_1_2_1_
Unit cell dimensions	*a* = 10.668(5) Å
	*b* = 13.475(5) Å
	*c* = 19.332(5) Å
Volume	2779.0(18) Å^3^
*Z*, Density (calc.)	4, 1.792 g/cm^3^
Absorption coefficient	3.441 mm^−1^
*F*(000)	1492
Crystal size	0.21 × 0.1 × 0.08 mm^3^
Completeness to theta = 25.00°	99.6%
Reflections (collected/independent)	18754/6025 [*R*(int) = 0.0217]
Data/restraints/parameters	6025/0/403
Goodness-of-fit on *F * ^2^	1.079
Final *R* indices [*I* > 2*σ*(*I*)]	*R *_1_ = 0.0193, *wR *_2_ = 0.0434
*R* indices (all data)	*R *_1_ = 0.0198, *wR *_2_ = 0.0437
Absolute structure parameter	0.599(6)
Largest diff. peak and hole	1.778 and −0.743 eÅ^−3^

**Table 2:** Selected bond distances (Å) and angles (°) for [Yb(NO_3_)_2_L](NO_3_)·2MeOH (4).

Bond distances					

Yb(1)–N(2)	2.410(3)	Yb(1)–O(6)		2.424(2)	
Yb(1)–O(4)	2.425(2)	Yb(1)–O(1)		2.428(2)	
Yb(1)–N(3)	2.433(3)	Yb(1)–O(2)		2.439(2)	
Yb(1)–O(7)	2.477(2)	Yb(1)–N(1)		2.531(3)	
Yb(1)–N(4)	2.538(2)	Yb(1)–O(3)		2.543(2)	
Yb(1)–N(6)	2.878(2)	Yb(1)–N(5)		2.909(3)	

Bond angles					

N(2)–Yb(1)–O(6)	72.51(8)	N(2)–Yb(1)–O(4)		116.04(8)	
O(6)–Yb(1)–O(4)	140.00(7)	N(2)–Yb(1)–O(1)		131.78(8)	
O(6)–Yb(1)–O(1)	76.60(8)	O(4)–Yb(1)–O(1)		69.55(7)	
N(2)–Yb(1)–N(3)	160.59(8)	O(6)–Yb(1)–N(3)		115.07(8)	
O(4)–Yb(1)–N(3)	70.63(8)	O(1)–Yb(1)–N(3)		67.34(8)	
N(2)–Yb(1)–O(2)	67.19(8)	O(6)–Yb(1)–O(2)		69.61(7)	
O(4)–Yb(1)–O(2)	78.14(8)	O(1)–Yb(1)–O(2)		67.75(8)	
N(3)–Yb(1)–O(2)	131.79(8)	N(2)–Yb(1)–O(7)		113.47(7)	
O(6)–Yb(1)–O(7)	52.03(7)	O(4)–Yb(1)–O(7)		129.66(8)	
O(1)–Yb(1)–O(7)	71.37(7)	N(3)–Yb(1)–O(7)		65.75(7)	
O(2)–Yb(1)–O(7)	114.18(7)	N(2)–Yb(1)–N(1)		65.39(8)	
O(6)–Yb(1)–N(1)	76.95(8)	O(4)–Yb(1)–N(1)		143.01(8)	
O(1)–Yb(1)–N(1)	139.88(8)	N(3)–Yb(1)–N(1)		98.00(9)	
O(2)–Yb(1)–N(1)	128.01(8)	O(7)–Yb(1)–N(1)		68.60(8)	
N(2)–Yb(1)–N(4)	98.28(8)	O(6)–Yb(1)–N(4)		145.03(7)	
O(4)–Yb(1)–N(4)	74.70(7)	O(1)–Yb(1)–N(4)		127.32(8)	
N(3)–Yb(1)–N(4)	64.96(9)	O(2)–Yb(1)–N(4)		138.96(7)	
O(7)–Yb(1)–N(4)	106.83(7)	N(1)–Yb(1)–N(4)		68.74(8)	
N(2)–Yb(1)–O(3)	67.07(8)	O(6)–Yb(1)–O(3)		131.84(7)	
O(4)–Yb(1)–O(3)	51.21(8)	O(1)–Yb(1)–O(3)		112.70(7)	
N(3)–Yb(1)–O(3)	111.73(8)	O(2)–Yb(1)–O(3)		71.32(7)	
O(7)–Yb(1)–O(3)	174.40(7)	N(1)–Yb(1)-O(3)		107.42(8)	
N(4)–Yb(1)–O(3)	67.71(7)	N(2)–Yb(1)–N(6)		93.83(7)	
O(4)–Yb(1)–N(6)	140.30(7)	O(1)–Yb(1)–N(6)		71.05(7)	
N(3)–Yb(1)–N(6)	90.01(8)	O(2)–Yb(1)–N(6)		91.46(7)	
N(1)–Yb(1)–N(6)	71.87(8)	N(4)–Yb(1)–N(6)		128.73(7)	
O(3)–Yb(1)–N(6)	57.89(7)	N(2)–Yb(1)–N(5)		91.86(8)	
O(6)–Yb(1)–N(5)	143.58(7)	O(1)–Yb(1)–N(5)		91.67(7)	
N(3)–Yb(1)–N(5)	90.64(8)	O(2)–Yb(1)–N(5)		74.03(7)	
O(7)–Yb(1)–N(5)	154.67(7)	N(1)–Yb(1)–N(5)		126.83(8)	
N(4)–Yb(1)–N(5)	68.13(7)	N(6)–Yb(1)–N(5)		160.92(7)	

Structural characteristics of H-bonds					

D–H⋯A		d(D–H)	d(H ⋯ A)	d(D ⋯ A)	<(DHA)
O(12)–H(4S) ⋯ O(11)		1.00(8)	1.89(8)	2.828(4)	156(7)
O(13)–H(8S) ⋯ O(12)#1		0.98(5)	1.79(5)	2.758(4)	170(5)

Symmetry transformations used to generate equivalent atoms: #1 –*x *+ 2, *y *− (1/2), −*z *+ (3/2).
